# Treatment of obesity with the resveratrol-enriched rice DJ-526

**DOI:** 10.1038/srep03879

**Published:** 2014-01-27

**Authors:** So-Hyeon Baek, Hea-Jong Chung, Heui-Kwan Lee, Roshan D'Souza, Youngju Jeon, Hyeon-Jin Kim, Soon-Jong Kweon, Seong-Tshool Hong

**Affiliations:** 1 National Institute of Crop Science, Rural Development Administration, Iksan, Chonbuk, Korea; 2 Department of Biomedical Sciences, Chonbuk National University Medical School, Jeonju, Chonbuk, Korea; 3 JINIS BDRD institute, JINIS Biopharmaceuticals Co., 948-9 Dunsan, Bongdong, Wanju 565-902, Jeollabuk-do, South Korea; 4 National Academy of Agricultural Science, Rural Development Administration, Suwon, Gyeonggi, Korea; 5 These authors contributed equally to this work.

## Abstract

Obesity is the most prevalent disease in the world which poses a serious risk for various chronic diseases. However, currently there are not any therapeutic agents that reduce body weight without causing serious side effects. In order to prevent and/or treat obesity and related diseases through a nutraceutical approach, we created a resveratrol-enriched transgenic rice accumulating 1.4 μg/g of resveratrol in its grain, DJ-526. Feeding of mice with the resveratrol-enriched rice DJ-526 showed excellent anti-obesity effect with reduction of body weights and abdominal fat volumes compared to the control by 20.0% and 31.3%, respectively. Also, the consumption of the resveratrol-enriched rice DJ526 significantly improved the blood lipid profiles and glucose levels in the animal experiments. Our resveratrol-enriched rice DJ-526 rice could provide both safe and convenient way for people with obesity and related diseases without major change of lifestyle or unwanted side effects from medication.

Obesity is a major health problem throughout the world, and it is increasing both in prevalence and severity^1^. According to the WHO, the current population of 1.6 billion overweight/obese people is expected to double by 2015. Pharmaceutical drugs such as orlistat, lorcaserin, sibutramine, phentermine, diethylpropion, or fluoxetine have been developed for the treatment of obesity^2^,^3^. However, benefits of these pharmaceutical drugs do not outweigh the side effects of the drugs in most cases^4^. Treatments of obesity with these drugs despite short-term benefits, is often associated with rebound weight gain after the cessation of drug use and serious side effects from the medication. As such, it is necessary to develop a new type of anti-obesity treatment that treats obesity without long-term side effects. Because obesity is caused by a nutrient-uptake imbalance where the amount of energy intake exceeds the amount of energy expended, one option to treat obesity effectively without side effect would be a nutraceutical approach^5^,^6^. Although some have been described to have dietary effects, the impact on obesity and related diseases are not enough to be claim its efficacy^7^. For an effective treatment, it would be reasonable to think that supplementation of nutrient ingredients having anti-obesity effect to an anti-obesity nutraceutical would generate a much more fortified anti-obesity nutraceutical.

Resveratrol (3,5,4′-trihydroxy-trans-stilbene) has been well recognized for its lipid-lowering function as well as calorie-restriction effect, proposing itself as one of the best anti-obese ingredients^8^,^9^,^10^,^11^. To generate more effective and convenient way for dietary consumption of resveratrol other than red wine, one of the major research efforts in plant science was to create transgenic cereal plants that accumulate resveratrol in their grains^12^,^13^. However, resveratrol was only detected at low levels in the leaves and stems except our previous work^14^. We previously created the resveratrol-enriched rice by transferring the resveratrol biosynthesis gene, stilbene synthase (*STS*) to Dongjin rice through the expression validation approach^15^. Among the 398 T_1_ small plantlets, one line of resveratrol-enriched rice plants, DJ-526, showed the excellent agricultural characteristic as well as accumulating a large quantity of resveratrol in its grain.

Parental plant of the resveratrol-enriched rice DJ-526, *Oryza sativa* japonica variety Dongjin rice developed by the Rural Development Administration of Korea, has characteristics of having grains rich in fiber and polyphenols with anti-obesity activity^14^. Thus, the resveratrol-enriched rice DJ-526 accumulating a large quantity of resveratrol in its grains in addition to fiber and polyphenols could be an ideal nutraceutical to treat obesity and its related diseases. The purpose of this research was to investigate whether the resveratrol-enriched rice DJ-526 is effective for obesity and its related diseases through a synergistic combination of the innate anti-obesity property of Dongjin rice and the lipid-lowering property of transgenic resveratrol. Our animal experiments showed that the resveratrol-enriched rice DJ-526 has strong anti-obesity effects and significantly improved all aspects of obesity-related diseases, suggesting its potential as an anti-obesity nutraceutical.

## Results

### The grains of the resveratrol-enriched rice DJ-526 contained a large amount of resveratrol

**Figure 1:**
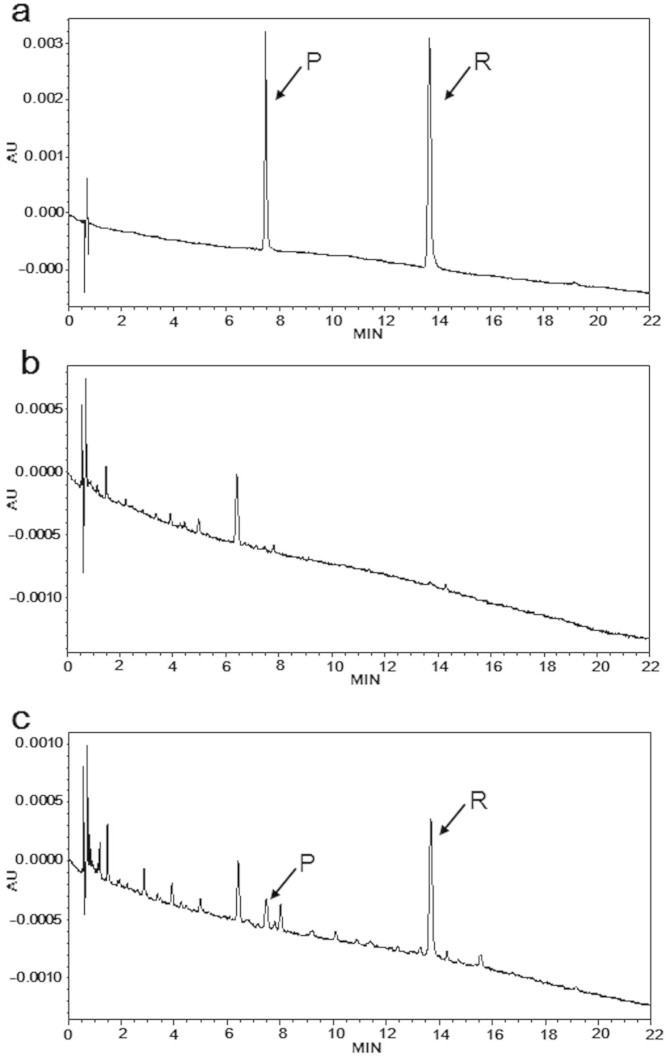
Identification of resveratrol and piceid in the grains of wild type Dongjin and the resveratrol-enriched rice DJ-526 by HPLC. (a) The HPLC profile of a standard mixture of piceid and resveratrol. (b) The HPLC profile of the grains of wild type Dongjin. (c) The HPLC profile of the grains of the resveratrol-enriched rice DJ-526. Arrows indicate piceid (P) and resveratrol (R) that correspond to each standard.

One of the current challenges in creating transgenic plants is to make a desired gene or genes functional specifically in the targeted part of the transgenic plants. It has been well known that piceid are accumulated as one of the major compounds in the plants having either natural or transgenic resveratrol biosynthetic pathways^16^. Because the health benefits of piceid are insignificant and long-term effect on the physiology of human has not been completely understood^17^,^18^, we aimed to select a transgenic rice plant accumulating mostly resveratrol rather than piceid in its grain. As we previously reported^15^, we first massively screened the resveratrol quantity of 398 T_1_ transgenic plants which contained the *AhSTS1*gene for resveratrol production. After identifying the candidate T_1_ transgenic plants, the candidate T_1_ transgenic plants were transplanted into a rice paddy. By the thorough analyses of the metabolic profile of the resveratrol and its related metabolites in the every portion of the transgenic plants, we selected a transgenic plant accumulating large quantity of resveratrol in its grain, DJ-526^15^. Figure 1 showed the metabolic profiles of resveratrol and the its related compound, piceid, from the resveratrol-enriched rice DJ-526 using HPLC. As expected, neither resveratrol nor piceid was detected in the HPLC analysis on the wild type Dongjin rice (Figure 1b). On the other hand, the grains of the resveratrol-enriched rice DJ-526 contained a relatively high quantity of resveratrol compared with piceid (Figure 1c), whereas the high quantity of piceid compared with resveratrol was observed in the leaves. In fact, resveratrol quantity in the grain of the resveratrol-enriched rice DJ-526, 1.4 μg/g, were close to the typical levels of resveratrol quantity in high-quality red wine, 0.8–5.8 μg/mL^19^. The preferential distribution of the two related metabolites, high accumulation of resveratrol in the edible grains but low in the leaves, makes the resveratrol-enriched rice DJ-526 ideal as a resveratrol-enriched cereal plant.

### The resveratrol-enriched rice DJ-526 rice showed an excellent anti-obesity effect by reducing body weights and abdominal fat volumes

**Figure 2:**
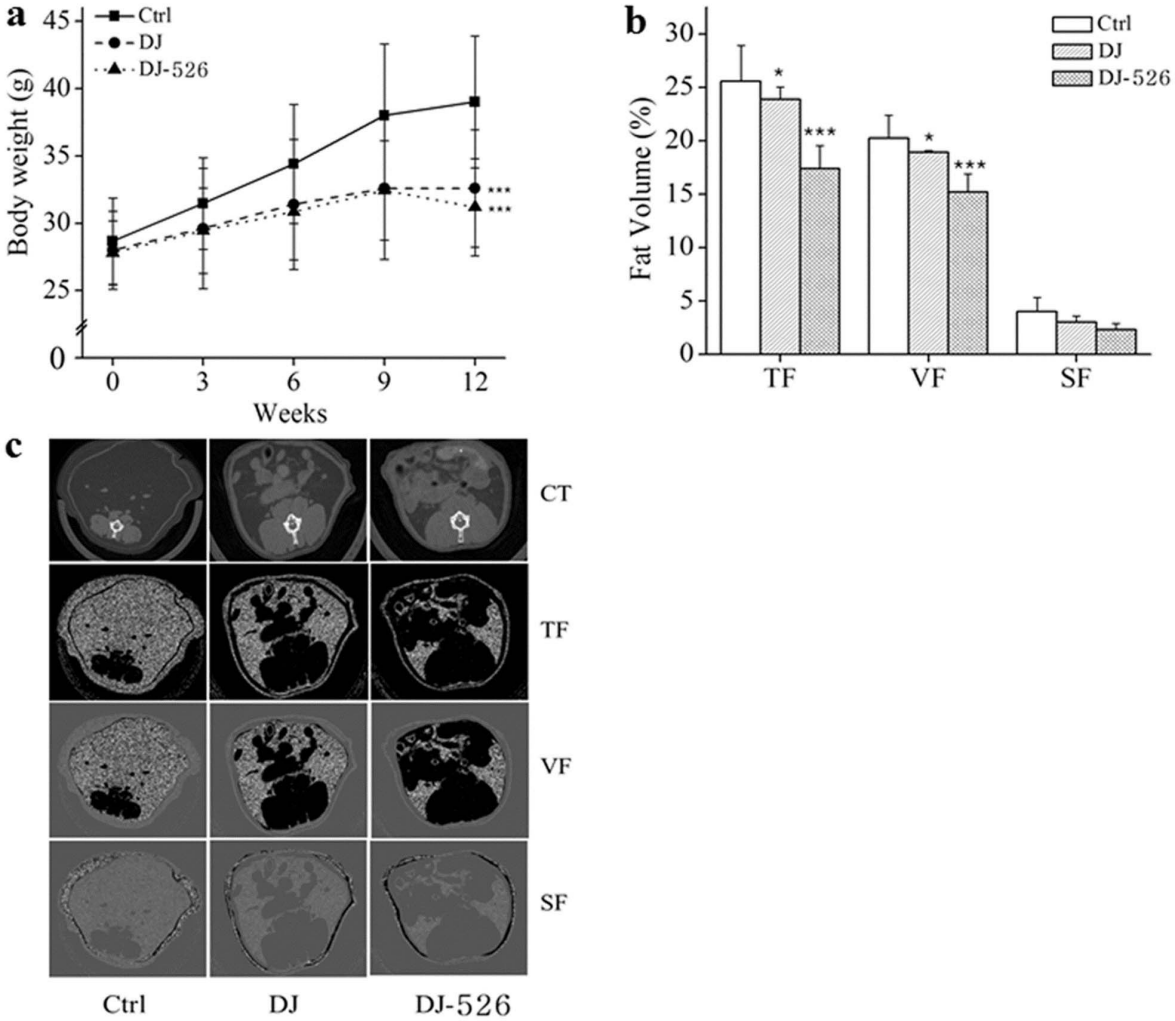
The effects of the resveratrol-enriched rice DJ-526 on changes in body weight and abdominal fat volume in mice with diet-induced obesity. (a) Changes in the body weight of mice during 12-week period. The values represent the means ± SEM (n = 30). An unpaired Student's t-test was used for the statistical analysis; ***p < 0.001 compared with Ctrl. (b) Changes in the fat volume of mice measured by *in vivo* micro-CT image analysis. The values represent the means ± SEM (n = 30). An unpaired Student's t-test was used for the statistical analysis; *p < 0.05, ***p < 0.001 compared with Ctrl. (c) The representative images of the micro-CT image analysis. TF, total fat; VF, visceral fat; SF, subcutaneous fat.

To investigate whether the resveratrol-enriched rice DJ-526 has an anti-obesity effect through a synergistic effect of the innate characteristics of Dongjin and the transgenic resveratrol as we expected, the efficacy of the resveratrol-enriched rice DJ-526 on obesity was examined using an *in vivo* mouse model. The C57BL/6 inbred mice with diet-induced obesity were fed the high fat diet (HFD) for 12 weeks in the control group or a modified HFD in the experimental group, in which the carbohydrate source was replaced with either Dongjin rice or the resveratrol-enriched rice DJ-526 (supplementary Table S1). Because body weight and abdominal fat volumes are the phenotypes of obesity, changes in the body weight and abdominal fat volumes in each mouse group were periodically monitored under the continued HFD conditions. The food consumption rate was the same among different mouse groups during the experimental period. Figure 2 displayed the changes in the body weight and abdominal fat volumes after dietary consumption of the resveratrol-enriched rice DJ-526. As shown in figure 2a, body weights were greatly reduced in mice fed with the resveratrol-enriched rice DJ-526 by 20.0% compared to the control, slightly greater than Dongjin rice group. More significant data came from micro-CT image analyses on abdominal fat deposition (Figure 2b). The total, visceral and subcutaneous fat volumes in the resveratrol-enriched rice DJ-526 group were 17.4%, 15.2% and 2.3%, respectively, which were significantly lower than the fat volumes from the control (25.6%, 20.2% and 4.0%, respectively) and the Dongjin group (23.9%, 18.9% and 3.0%, respectively). The representative images of the micro-CT image analyses on abdominal fat deposition clearly indicated that the total, visceral and subcutaneous fat accumulation was the lowest in the resveratrol-enriched rice DJ-526 group compared with the other treatments (Figure 2c). Based on the experimental results of the efficacy of the resveratrol-enriched rice DJ-526 on body weight and abdominal fat volumes, it was concluded that the resveratrol-enriched rice DJ-526 had an excellent anti-obesity effect.

### The consumption of the resveratrol-enriched rice DJ-526 significantly improved lipid profiles and blood glucose levels with clear anti-obesity effect in animal experiments under high-fat diet

**Figure 3:**
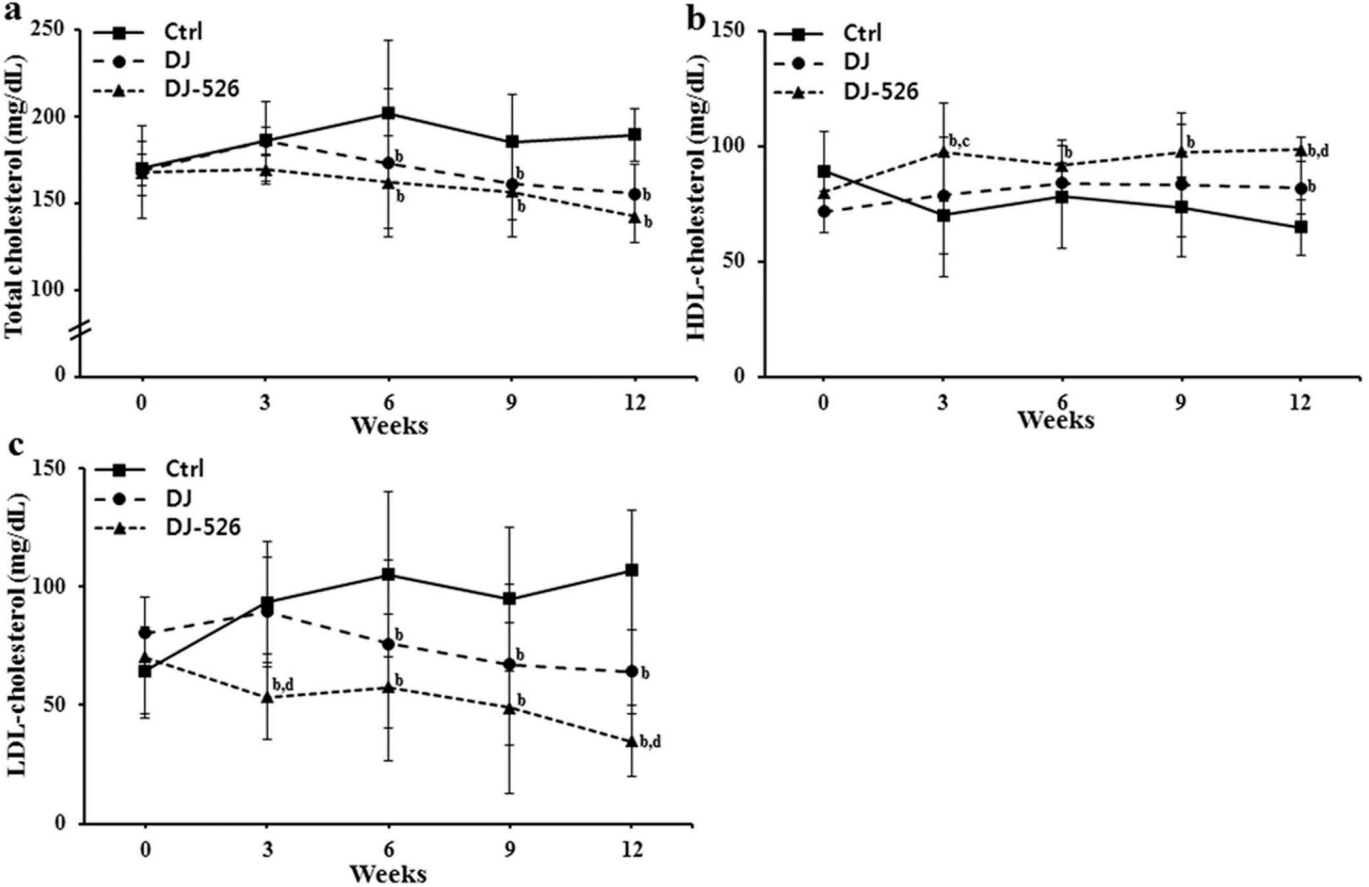
The effects of the resveratrol-enriched rice DJ-526 on changes in blood cholesterol profiles in mice with diet-induced obesity. The effects of the resveratrol-enriched rice DJ-526 on blood total cholesterol (a), HDL-cholesterol (b) and LDL-cholesterol (c) of mice during the 12-week experimental period. The values represent the means ± SEM (n = 30). Values in the figure with a superscripted letter indicate statistical significance as analyzed by an unpaired Student's t-test; (a): p < 0.05 compared with Ctrl; (b): p < 0.01 compared with with Ctrl; (c): p < 0.05 compared with Dongjin; (d): p < 0.01 compared with Dongjin. Ctrl, mice fed a HFD; Dongjin, mice fed a HFD in which the corn starch and sucrose was replaced with Dongjin rice; DJ-526, mice fed a HFD on which the corn starch and sucrose were replaced with the resveratrol enriched rice DJ-526.

**Figure 4:**
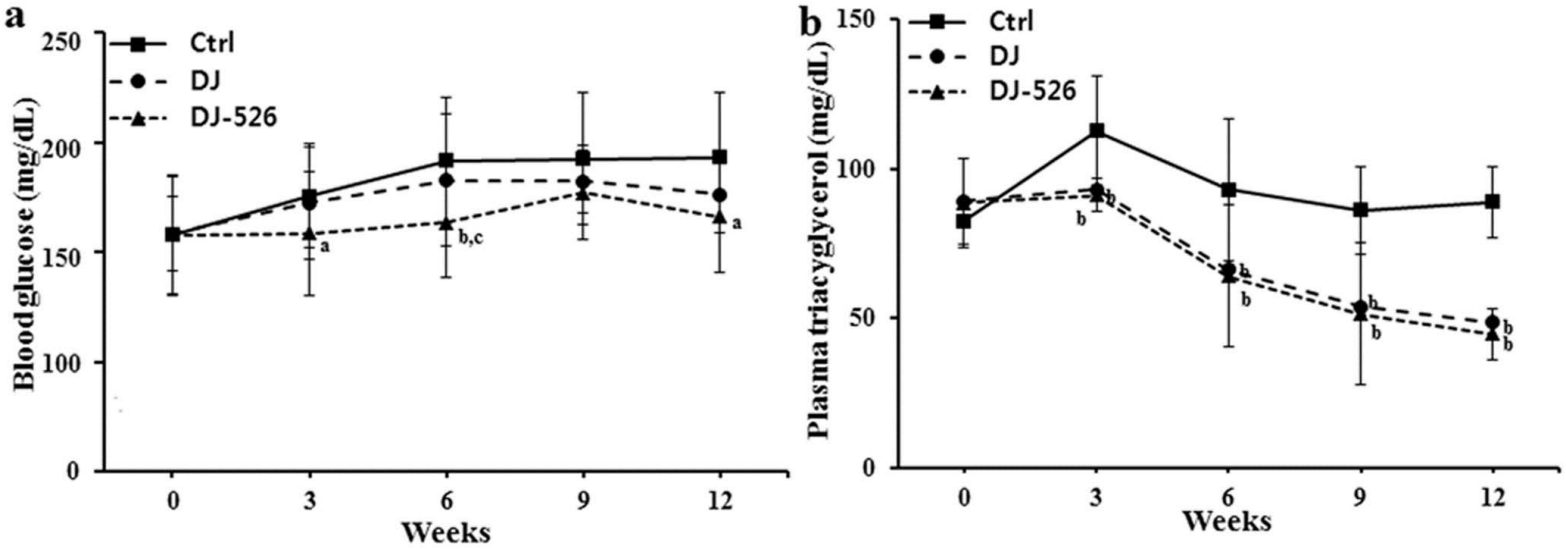
The effects of the resveratrol-enriched rice DJ-526 on changes in blood glucose and plasma triacylglycerol in mice with diet-induced obesity. The effects of the resveratrol-enriched rice DJ-526 on the blood glucose (a) and plasma triacyglycerol level (b) of mice during the 12-week experimental period. The values represent the means ± SEM (n = 30). Values in the figure with a superscripted letter indicate statistical significance as analyzed by an unpaired Student's t-test; (a): p < 0.05 compared with Ctrl; (b): p < 0.01 compared with Ctrl; (c): p < 0.05 compared with Dongjin; (d): p < 0.01 compared with Dongjin. Ctrl, mice fed a HFD; Dongjin, mice fed a HFD in which the corn starch and sucrose was replaced with Dongjin rice; DJ-526, mice fed a HFD on which the corn starch and sucrose were replaced with the resveratrol enriched rice DJ-526.

Obesity is not simply a consequence of overweight but is typically complicated by diabetes and hyperlipidemia. Because the nature of obesity, we examined the efficacy of the resveratrol-enriched rice DJ-526 on the obesity-related diseases by monitoring changes in the levels of blood glucose, triacylglycerol, total cholesterol, HDL-cholesterol, LDL-cholesterol using an *in vivo* mouse model. The consumption of Dongjin rice resulted in some improvements in lipid profile and blood glucose levels compared to the control, as expected from its endogenic nature. More importantly, the consumption of the resveratrol-enriched rice DJ-526 significantly improved the lipid profiles as well as blood glucose. The consumption of the resveratrol-enriched rice DJ-526 lowered total cholesterol by 17.9%, and LDL-cholesterol by 67.3%, while increasing HDL-cholesterol by 34.2% compared to the control (Figure 3). Also, the consumption of the resveratrol-enriched rice DJ-526 lowered blood glucose by 13.9% and triacylglycerol by 49.3% compared to the control (Figure 4).

## Discussion

Obesity is complicated with various diseases such as diabetes, hypercholesterolemia, hyperlipidemia, metabolic syndrome, *etc*^1^. Currently, obesity and its related diseases became a major health problem throughout the world that is increasing both in prevalence and severity^20^. In spite of serious efforts to treat and prevent obesity and its related diseases, an ideal solution for obesity has not been developed. Considering efficacy and their side effects, current pharmaceutical drugs to treat obesity and its related diseases do not provide a solution for obesity and its related diseases so that only 6% of obese patients are treated pharmacologically^21^. We believe that the resveratrol-enriched rice DJ-526 is an ideal nutraceutical solution to treat or prevent obesity. The anti-obesity effect resulting from the synergistic effect of Dongjin rice and resveratrol showed efficacy levels aiming to treat obesity and its related diseases as much as the typical pharmaceutical drugs. Current pharmaceutical drugs only target individual aspects of obesity and its related diseases, such as blood glucose, LDL/total cholesterol, or body weight. There is no pharmaceutical drug available to treat every aspect of obesity and its related diseases. We believe that the resveratrol-enriched rice DJ-526 could be an ideal choice to target most, if not all, aspects of obesity and its related diseases.

Due to the synergistic effect of the endogenous anti-obesity effect of the Dongjin rice and the lipid-lowering effect of resveratrol, the resveratrol-enriched rice DJ-526 has more potent anti-obesity activity than Dongjin rice itself so that the resveratrol-enriched rice DJ-526 can be used to treat and prevent obesity and its related diseases. Both the severity and prevalence of obesity and its related diseases, such as cardiovascular diseases, and diabetes, among many others, are more serious in developing countries than in developed countries. Limited medical care in developing countries make obesity and its related diseases is a more serious issue in developing countries than in developed countries. We believe that the resveratrol-enriched rice DJ-526 could be an excellent alternative for the management of obesity and its related diseases, not only in developed countries but also in developing countries.

Finally our work provides an inspiration for a future development of genetically modified crops. This work showed that a synergistic effect of the innate property of a host plant and a transgenic property significantly augmented the original anti-obesity property of the Dongjin rice. We believe that a careful selection of host plant and transgene would lead to create a very interesting functional crop to manage many chronic diseases.

## Methods

### Quantification of resveratrol and piceid

The eight-week-old leaves and mature grains of the wild-type cultivar Dongjin and the resveratrol-enriched rice DJ-526 rice plants were used to determine the levels of resveratrol and the related resveratrol glucoside piceid as described previously^15^. After the fresh samples of the leaves and grains were freeze-dried, the samples were homogenized to be extracted with 80% ethanol. 1 μL of the each sample was analyzed by a reverse phase HPLC equipped with a UV detector under the gradient condition (ACQUITY TUV, Waters, Milford, USA). The ACQUITY UPLC BEH-C18 1.7 μm column (2.1 mm × 100 mm, Waters) was used at a flow rate of 0.4 mL/min. The mobile phase was 10 to 90% acetonitrile (ACN). A gradient elution was conducted as follows: 0 min, 10% ACN; 1.54 min, 10% ACN; 10 min, 15% ACN; 22 min, 25% ACN; 22.4 min, 90% ACN; and 25 min, 90% ACN; followed by re-equilibration of the column with 10% ACN for 5 min prior to the next injection. Each metabolite peak of samples and standard chemical samples was calculated using the Empower software (Waters) to determine quantity of the metabolites. The HPLC fractions of resveratrol and piceid were further verified using GC-MS analysis with the 6890/5973N GC/MS system (Agilent Technologies, Santa Clara, CA, USA) equipped with an Rtx-5MS capillary column (30 mm × 0.25 mm I.D., 0.25 μm film thickness).

### Animal experiments

Six-week-old female C57BL/6 mice (Joongang Experimental Animal Co., Seoul, Korea) were purchased and acclimatized for 2 weeks. Then, the animals were fed with a high-fat diet (HFD) containing 45% calorie as lard fat (D12451, Research Diets Inc., New Brunswick, NJ, USA) for 12 weeks. Mice with diet-induced obesity and related diseases were randomly divided and fed the HFD, modified HFD in which the corn and sucrose were replaced with Dongjin rice, and modified HFD in which the corn and starch were replaced with the resveratrol-enriched rice DJ-526. Food intake and body weight were measured regularly, and blood samples were taken at indicated time points. Fasting blood glucose was measured using Accu-check Glucometer (Roche, Indianapolis, IN, USA) and lipid profile analysis was performed using as enzymatic colorimetric method (Asan Pharm., Yongjin, Korea). Fat volume was analyzed from anesthetized mice by high-resolution *in vivo* micro-CT (Skyscan 1076; Skyscan, Konitech, Belgium). The total fat, visceral fat and subcutaneous fat areas were analyzed with Micro-CT images at the level of the L1-L5 intervertebral disk using CTan Ver.1.10 software (Skyscan).

All animal care and use were performed strictly in accordance with the ethical guidelines by the Ethics Committee of Chonbuk National University Laboratory Animal Center, and the animal study protocol was approved by the institution.

## Author Contributions

S.B., H.C., H.L., R.D. and Y.J. performed the experiments. H.K., S.K. and S.H. designed experiments, analyzed and interpreted the data, and wrote the manuscript.

## Supplementary Material

Supplementary Information Supplementary Table S1
