# Supplementary material for: Treatment of obesity with the resveratrol-enriched rice DJ-526
Source: Sci Rep. 2014 Jan 27;4:3879. doi: 10.1038/srep03879 (PMC3902431; doi:10.1038/srep03879)
Supplement: Supplementary Information — Supplementary Table S1 [file srep03879-s1.pdf]

Supplementary Information

Treatment of obesity with the resveratrol-enriched rice DJ-526

So-Hyeon Baek[1,5], Hea-Jong Chung[2,5], Heui-Kwan Lee[2,5], Roshan D'Souza[2], Youngju Jeon[2], Hyeon-Jin Kim[3], Soon-Jong Kweon[4] & Seong-Tshool Hong[2*]

[1] National Institute of Crop Science, Rural Development Administration, Iksan, Chonbuk, Korea

[2] Laboratory of Biomedical Sciences, Chonbuk National University Medical School, Jeonju, Chonbuk, Korea

[3] JINIS BDRD institute, JINIS Biopharmaceuticals Co., 948-9 Dunsan, Bongdong, Wanju 565-902, Jeollabuk-do, South Korea.

[4] National Academy of Agricultural Science, Rural Development Administration, Suwon, Kyunggi, Korea

∗Correspondence: ST. Hong, Department of Biomedical Sciences, Chonbuk National University Medical School, Keumam-Dong San 2-20, Chonju, Chonbuk 561-180, Korea

E-mail: seonghong@chonbuk.ac.kr

[5]These authors contributed equally to this work.

Supplementary Table S1. The formulation of the animal diets (g)

Ingredient	CTL (D12451)	Dongjin	DJ-526
Casein, 80 Mesh	200	200	200
L-Cystine	3	3	3
**Corn Starch**	**72.8**	**0**	**0**
Maltodextrin 10	100	100	100
**Sucrose**	**172.8**	**0**	**0**
Cellulose, BW200	50	50	50
Soybean Oil	25	25	25
Lard	177.5	177.5	177.5
Mineral Mix	10	10	10
Dicalcium Phosphate	13	13	13
Calcium Carbonate	5.5	5.5	5.5
Potassium Citrate	16.5	16.5	16.5
Vitamin Mix	10	10	10
Choline Bitartrate	2	2	2
FD&C Red Dye #40	0.05	0.05	0.05
**Dongjin Rice**	**0**	**245.6**	**0**
**DJ-526 Rice**	**0**	**0**	**245.6**
Total	858.15	858.15	858.15

* Deviations from the control HFD (D12451) are indicated with bold.

After manufacturing the diets, we reconfirmed the quantity of resveratrol in each diet using HPLC before feeding the animals.
